# The roles of Th cells in myocardial infarction

**DOI:** 10.1038/s41420-024-02064-6

**Published:** 2024-06-15

**Authors:** Jun Liu, Feila Liu, Tingting Liang, Yue Zhou, Xiaohan Su, Xue Li, Jiao Zeng, Peng Qu, Yali Wang, Fuli Chen, Qian Lei, Gang Li, Panke Cheng

**Affiliations:** 1 School of Pharmacy and Bioengineering, Chongqing University of Technology, Chongqing, China; 2 Department of Breast and Thyroid Surgery, Biological Targeting Laboratory of Breast Cancer, Academician (expert) workstation, Affiliated Hospital of North Sichuan Medical College, Nanchong, China; 3 Department of Laboratory Medicine, Affiliated Hospital of North Sichuan Medical College, Nanchong, China; 4 Institute of Cardiovascular Diseases & Department of Cardiology, Sichuan Provincial People’s Hospital, School of Medicine, University of Electronic Science and Technology of China, Chengdu, China; 5 Department of Anesthesiology, Sichuan Provincial People’s Hospital, School of Medicine, University of Electronic Science and Technology of China, Chengdu, China; 6 Ultrasound in Cardiac Electrophysiology and Biomechanics Key Laboratory of Sichuan Province, Chengdu, China

**Keywords:** Myocardial infarction, Acute inflammation

## Abstract

Myocardial infarction, commonly known as a heart attack, is a serious condition caused by the abrupt stoppage of blood flow to a part of the heart, leading to tissue damage. A significant aspect of this condition is reperfusion injury, which occurs when blood flow is restored but exacerbates the damage. This review first addresses the role of the innate immune system, including neutrophils and macrophages, in the cascade of events leading to myocardial infarction and reperfusion injury. It then shifts focus to the critical involvement of CD4+ T helper cells in these processes. These cells, pivotal in regulating the immune response and tissue recovery, include various subpopulations such as Th1, Th2, Th9, Th17, and Th22, each playing a unique role in the pathophysiology of myocardial infarction and reperfusion injury. These subpopulations contribute to the injury process through diverse mechanisms, with cytokines such as IFN-γ and IL-4 influencing the balance between tissue repair and injury exacerbation. Understanding the interplay between the innate immune system and CD4+ T helper cells, along with their cytokines, is crucial for developing targeted therapies to mitigate myocardial infarction and reperfusion injury, ultimately improving outcomes for cardiac patients.

## Facts

- CD4+ Th cells play a critical role in the immune-inflammatory responses and cardiac remodeling during myocardial infarction and reperfusion injury. They may have dual roles, significantly impacting cardiac function and prognosis.
- Different subsets of CD4+ Th cells (such as Th1, Th2, Th9, Th17, and Th22) secrete various cytokines and play distinct roles in inflammation, anti-inflammation, and remodeling/repair following myocardial infarction and reperfusion. The balance among these subsets is crucial for prognosis.
- The application of immune regulatory therapies targeting CD4+ Th cells in myocardial infarction and reperfusion injury is still in the early stages of research.
- Effective regulation of CD4+ Th cell functions require the coordinated action of multiple cell types, making comprehensive immune response regulation highly challenging and uncertain.

## Open Questions

- Can further research determine why the same subtype of CD4+ Th cells exhibit different functions under different conditions?
- When multiple cells express the same cytokines, how can we accurately identify the primary contributors?
- Can we explore and regulate key immune cells to achieve effective control of the overall immune response, thereby protecting the myocardium?

## Introduction

Acute myocardial infarction (AMI) is characterized by myocardial tissue necrosis resulting from unstable manifestations of ischemic syndromes [1]. The pathogenesis of AMI typically involves the reduction of coronary blood flow, precipitating myocardial infarction. This process often stems from the rupture or erosion of vulnerable, lipid-rich atherosclerotic coronary plaques, allowing blood to interact with hyperthrombotic core and matrix components within the plaque region [2]. Complete closure due to thrombus and plaque typically manifests as ST-segment elevation myocardial infarction (STEMI) [3]. Conversely, partial closure, or closure in the presence of collateral circulation, can lead to non-ST-segment elevation myocardial infarction (non-STEMI) or unstable angina pectoris [4]. In the context of ischemic heart disease (IHD) epidemiology in China, a notable trend emerges wherein the disease burden is escalating rapidly. Factors such as advancing age and unhealthy lifestyles, including smoking and poor dietary habits, contribute significantly to this rise. Moreover, hypertension, elevated cholesterol levels, and diabetes play direct roles in exacerbating IHD [5].

Timely reperfusion stands as the cornerstone for salvaging ischemic myocardium, with thrombolytic therapy or direct percutaneous coronary intervention (PCI) emerging as the most effective therapeutic interventions. These strategies prove instrumental in reducing acute myocardial ischemic injury and limiting myocardial infarction by promptly and efficiently restoring blood flow to the infarcted area [6]. However, it is crucial to acknowledge that while reperfusion holds the potential to salvage ischemic myocardium, it also poses the risk of causing irreversible damage, known as ischemia/reperfusion injury (I/RI) [7]. Several factors contribute to this damage, including oxidative stress, calcium overload, rapid physiological pH recovery, and inflammation. These factors play crucial roles in mediating cardiomyocyte death by inducing cardiomyocyte hypercontraction and opening of the Mitochondrial Permeability Transition Pore (MPTP). Specifically, after myocardial reperfusion, the acidic conditions induced by prior myocardial ischemia undergo a rapid recovery of physiological pH, the restoration of oxygen supply and inflammation combine to cause oxidative stress, and calcium overload due to myocardial membrane damage, all of which may lead to MPTP opening and myocardial hypercontraction, thereby causing further cardiomyocyte death. Notably, the opening of MPTP is responsible for cardiomyocyte death by uncoupling oxidative phosphorylation [8]. Hence, while reperfusion holds the potential to salvage ischemic myocardium, it can also lead to adverse consequences. Reperfusion injury manifests in four main categories: Firstly, reperfusion arrhythmias, typically resolving spontaneously or with minimal intervention. Secondly, myocardial tonicity, resulting from the deleterious effects of oxidative stress and intracellular calcium overload on myocardial contraction. Thirdly, microvascular obstruction, associated with a substantial infarcted region of the heart, reduced left ventricular ejection fraction, and potential adverse left ventricular remodeling, possibly leading to intramyocardial bleeding. Fourthly, fatal myocardial reperfusion injury, where reperfusion itself may contribute to 50% of the overall myocardial infarction area [9].

The process of myocardial infarction repair unfolds through three phases: the inflammatory phase, anti-inflammatory repair phase, and remodeling phase [10]. During myocardial ischemia and reperfusion, cell death, including that of vascular endothelial cells and cardiomyocytes, leads to the release of substantial cellular contents. These contents, termed endogenous ligands, act as “danger signals” during reperfusion. Recognition of these ligands by pattern-recognition receptors (PRRs), such as toll-like receptors (TLRs), triggers the activation of the nuclear factor (NF)-κB and mitogen-activated protein kinase (MAPK) pathways, culminating in the expression of pro-inflammatory cytokines, chemokines, and adhesion molecules, regulating the complex post-ischemic inflammatory response [11]. Furthermore, in response to danger-associated molecular patterns (DAMPs) released during myocardial ischemia/reperfusion (MI/R) and the liberation of cardiomyocyte contents during AMI, the complement cascade is activated. This cascade generates potent neutrophil chemotactic agents and upregulates adhesion molecules on endothelial cells, facilitating neutrophil migration [12]. Consequently, inflammation attracts additional phagocytes, perpetuating the cell-killing membrane-attacking complex and exacerbating MI/R-induced inflammation and injury [13]. This cascade of events recruits various inflammation-related cells, including neutrophils, monocytes, macrophages, B lymphocytes, and T lymphocytes, to the infarcted area, intensifying the pro-inflammatory response following MI/R. During the inflammatory phase, neutrophils and pro-inflammatory monocytes endeavor to clear necrotic cell debris from the myocardial infarction area. Failure to remove debris may precipitate the no-reflow phenomenon, potentially leading to new blockages. In the anti-inflammatory repair phase, alterations in monocyte and macrophage phenotypes occur, with contributions from anti-inflammatory CD4+ T cells and dendritic cells (DCs), promoting inflammation containment, wound healing, scar formation, and preventing cardiac rupture. Debris clearance is pivotal in averting no-reflow, with the anti-inflammatory repair phase playing a central role in cardiac healing. The final remodeling phase encompasses extracellular matrix (ECM) remodeling and neovascularization. Distinct immune cells undertake specific functions during each phase. Understanding these cellular functions can inform the development of tailored treatments to improve prognosis following AMI and reperfusion.

## Immune cells in MI

### Myeloid cells

The innate immune system comprises various myeloid cells, such as monocytes, macrophages, dendritic cells, natural killer (NK) cells, and neutrophils [14]. However, limited studies have directly explored myocardial leukocyte recruitment post-myocardial infarction (MI) in humans, with most data derived from mouse models. Following activation of the inflammatory pathway, these myeloid cells play pivotal roles in post-MI inflammation. Neutrophils, for instance, infiltrate the infarcted myocardium post-MI, worsening inflammation and damage (N1 phenotype), while an anti-inflammatory response (N2 phenotype) develops over time, inhibiting further inflammation [10]. Monocytes also quickly migrate to the reperfusion zone, differentiating into macrophages that replace tissue-resident macrophages, which aiding cardiac recovery [15]. This process involves two phases: initially, pro-inflammatory monocytes transition to M1 macrophages, promoting adverse left ventricular remodeling [11], followed by Ly6Clow monocytes transitioning to M2 macrophages, which prevent adverse remodeling [16]. NK cells also play a role in MI, with their absence during MI limiting apoptosis and regulating post-infarction myocardial fibrosis to a lesser extent [17]. NK cells and inflammatory macrophages may interact, mutually enhancing activity and inflammation in the infarcted area [18]. Additionally, dendritic cells (DCs), which can differentiate from Ly6Clow monocytes [19], migrate and accumulate in the injured myocardium post-MI/R. Several studies have highlighted the direct immunoprotective role of DCs in this context [20]. Moreover, DCs indirectly prevent adverse LV remodeling by recruiting CD4+ Th cells to the MI zone through exosome production [21].However, limiting DCs activation and migration also reduced infarct size [22].

### Lymphoid cells

In addition to the innate immune cells mentioned above, cells associated with adaptive immunity are actively involved in various mechanisms following MI/R. The central cellular components of adaptive immunity are B cells and T cells derived from lymphoid progenitors in the bone marrow. After AMI, the infiltration of mature B lymphocytes into the infarcted area of the myocardium induces pro-inflammatory monocytes to amplify the inflammatory response, thereby decreasing myocardial contractility, promoting apoptosis, and worsening left heart function [23]. However, it has also been shown that rats with intramyocardial injection of B cells reduced cardiomyocyte apoptosis and preserved left heart function, suggesting that B cells have a beneficial effect on myocardial protection after MI [24]. In addition, as B cells accumulate after MI, they preferentially produce interleukin-10 (IL-10), which facilitates the reduction of inflammation, attenuates myocardial injury, and protects cardiac function [25]. After 90 min of reperfusion, circulating T cells (predominantly CD8+ T cells) were reduced, a phenomenon that may be due to cell recruitment to ischemic cardiac tissue, which was most evident in patients with microvascular obstruction on cardiac MI/R injury [26]. CD8+ T cells can amplify inflammation by direct cytotoxicity to healthy cardiomyocytes, activate macrophage-mediated clearance of necrotic debris, and also induce and also lead to deterioration of cardiac function by degranulation [27]. However, by removing fibroblast activating proteins, CD8+ T cells also reduced cardiac fibrosis and improved cardiac function after injury in mice [28]. In addition, a specific subpopulation of CD8+ T cells expands in response to ischemic myocardial injury. These CD8+ T cells produce IL-10 in response to angiotensin II stimulation without exhibiting cytotoxicity and thus exert cardioprotective effects [10].

In addition to CD8+ T cells, CD4+ T cells play a complex and critical role after MI. Transcoronary gradient data in human patients suggest that CD4+ T cells infiltrate the reperfused myocardium within 45 min of reperfusion [26]. Activation of CD4+ T lymphocytes, possibly in response to cardiac autoantigens released after infarction, contributes to amelioration of adverse remodeling and improved survival [29]. This beneficial regulation of myocardial injury repair may be due to the fact that CD4+ T cells promote myocardial healing by influencing monocyte recruitment and differentiation. However, the absence of interferon-γ (IFN-γ) -releasing CD4+ T cells has been shown to reduce infarct size and preserve ejection fraction after MI/R [30]. CD4+ T cells are immune cells with numerous subtypes, each serving a distinct function. This variety in subtype function offers a potential explanation for the conflicting results.

Regulatory T (Treg) cells comprise a vital, albeit minor, subdivision of CD4+ T cells. Primarily, Treg cells release transforming growth factor (TGF) -β and IL-10 to restrain activation and proliferation of Th1 and Th2 lymphocytes and suppress innate immune cell effector mechanisms. Consequently, this serves to mitigate inflammation, autoimmune disorders, and alter the cytokine environment [31, 32]. Treg cells mediate myocardial repair and promotes stable scar formation [33]. Treg inhibits recruitment of inflammatory cells and suppresses the local expression of pro-inflammatory cytokines [33]. In the context of MI/R, Treg cells have been demonstrated to prevent cardiomyocyte apoptosis, minimize additional damage, and down-regulate fibroblast differentiation to myofibroblasts. By doing so, they evade pathological scar formation [34]. In conclusion, Treg cells seem to promote the whole process of wound healing while inhibiting pathological scar formation and playing a protective role after reperfusion in myocardial infarction.

In addition to classical CD4+ αβ T cells, γδT cells are involved in reperfused myocardium. In reperfusion myocardium, γδT cells contribute to interleukin-17A (IL-17A) secretion.IL-17A mediates cardiomyocyte apoptosis and increased neutrophil infiltration by increasing chemokines. Inactivation of IL-17A leads to reduced infarct size and improved ventricular function [35]. In addition to promoting increased infiltration of inflammatory cells, IL-17A also promotes fibrosis, resulting in unfavorable cardiac remodeling [36, 37]. However, another study have demonstrated that γδT cells secrete anti-inflammatory cytokines, such as TGF-β and IL-10, which stabilize the acute Th1 inflammatory response and suppress inflammation [38]. Invariant natural killer T (iNKT)cells can be activated through cytokines or antigens presented by CD1d, an MHC class I molecule. Studies have shown augmented infiltration of iNKT cells in the heart following a myocardial infarction, and further research has demonstrated that αGC-activated iNKT cells mitigate MI/R injury through increased expression of IL-10 [35]. In addition to the aforementioned cells, there are CD4+ helper T cells that require attention. Further division of these CD4+ T-cell subpopulations is needed to fully understand their role in MI/R and study their mechanism of action.

**Fig. 1:**
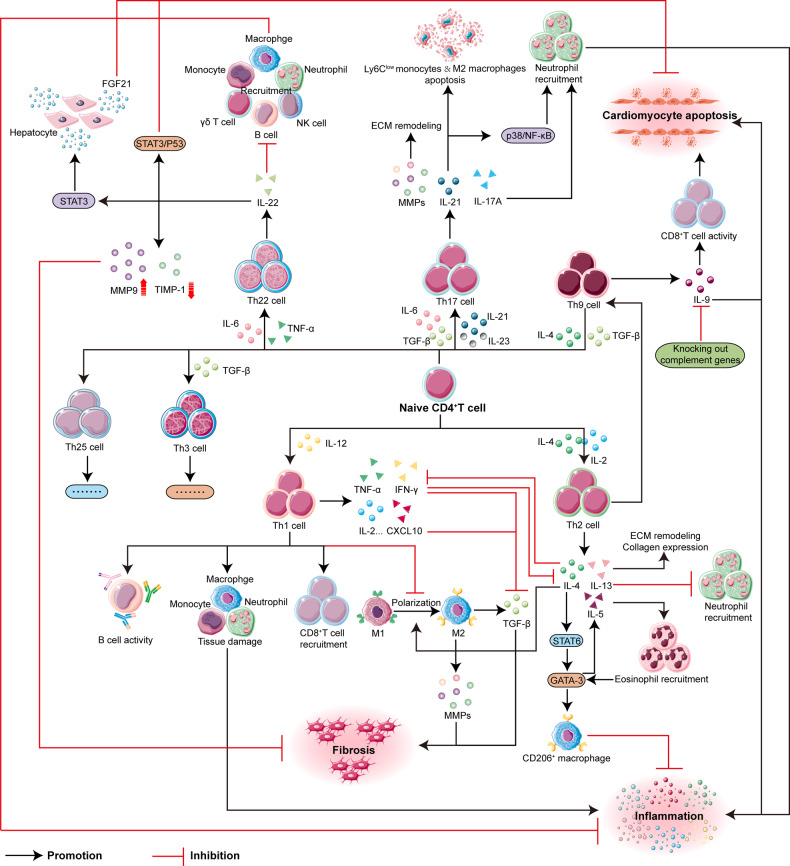
Overview of the lymphoid and myeloid cells’ role after reperfusion of myocardial infarction is depicted in this figure. It illustrates the pro-inflammatory and subsequent anti-inflammatory phases of repair and remodeling after reperfusion in myocardial infarction. The accumulation of multiple cells, including neutrophils, monocytes, macrophages, NK cells, dendritic cells, B lymphocytes, and CD8+ T cells, infiltrate the infarct zone. These cells exert pro-inflammatory, anti-inflammatory, or differential effects on fibrosis and pro-angiogenesis.

In summary, the active participation of immune cells plays a pivotal role in the reparative processes following MI. The intricate dynamics of immune cell distribution and interactions are visually represented in Fig. 1.

## T helper (Th) cells

**Fig. 2:**
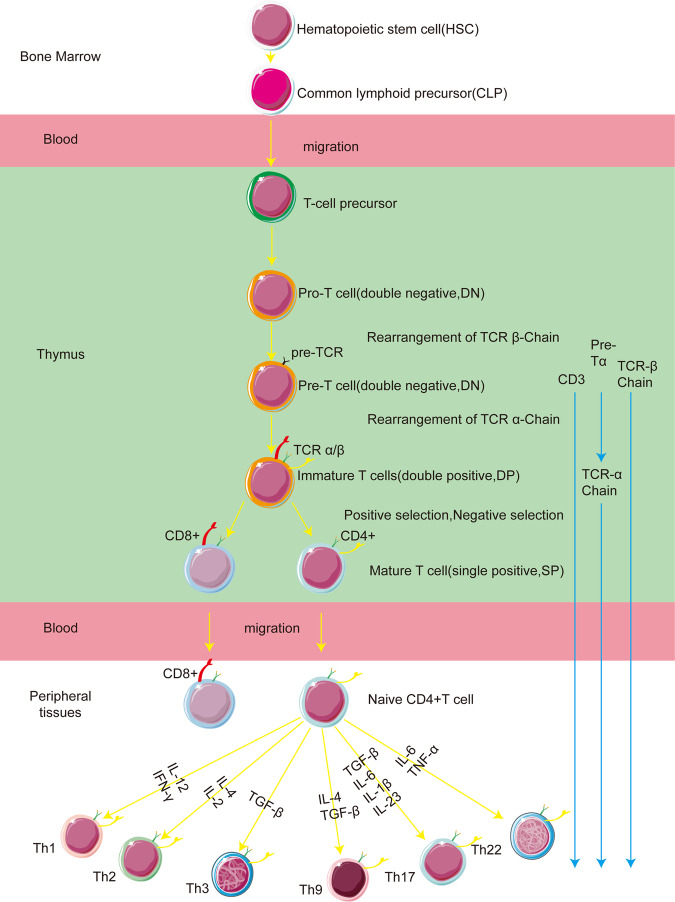
A schematic representation of the process of T-cell development. The diagram illustrates the differentiation of T cells from HSCs into distinct subtypes of Th cells. The development of Th cells begins with HSCs in the bone marrow, differentiating into CLPs. These CLPs migrate to the thymus, undergoing selection to become mature CD4+ T cells. The mature CD4+ T cells then travel to peripheral lymphoid organs (e.g., lymph nodes and spleen), where they are activated by antigens presented by APCs. This activation involves interactions between MHC class II and co-stimulatory molecules on APCs with TCR and CD28 on T cells, leading to their differentiation into various Th cell subtypes based on environmental signals.

T helper cell development originates from hematopoietic stem cells (HSCs) in the bone marrow, which differentiate to give rise to multipotent hematopoietic progenitors (MAPs). These progenitors then undergo further differentiation into common lymphoid precursor cells (CLPs). These CLPs migrate to the thymus, where they undergo both positive and negative selection processes to eventually generate mature CD4+ T cells (NAIVE CD4+ T cells). Mature CD4+ T cells migrate through the circulation to peripheral lymphoid organs (e.g., lymph nodes and spleen) where they encounter antigens presented by antigen-presenting cells (APCs). Initial activation is accomplished through the interaction of MHC class II molecules and co-stimulatory molecules (e.g., CD80/CD86) of APCs with TCR and CD28 molecules on the surface of CD4+ T cells. Subsequently, they can differentiate into various Th cell subtypes, in response to different stimuli and microenvironmental signals (as shown in Fig. 2).

Activation of T cells following MI increases 5–10 times in hearts with MI/R at day 7 compared to healthy controls, driven by recognition of cardiac antigens in a mouse reperfusion model [39]. These cells have multiple phenotypes and play an immune role in activating other immune cells that are involved in regulating MI [40]. CD4+ helper T cells comprise various subtypes, including Th1, Th2, Th9, Th17, Th22, and others, which impact immune response and myocardial repair by secreting diverse cytokines and regulatory mechanisms. These subtypes play distinct roles in I/RI and its subsequent repair phase, which cumulatively have an impact on the final recovery outcome. For example, during the inflammatory phase of MI, a Th1 immune response induced by Th1 cells helps clear necrotic debris. However, this response can also exacerbate the damage associated with MI [41]. During the healing process, the Th1-mediated immune response is reduced and replaced by Th2-mediated immunity, during which leukocytes alter their phenotype to coordinate tissue regeneration and prevent cardiac rupture [13]. However, a robust Th2 response may cause pathological scarring, emphasizing the significant reliance of this occurrence on highly precise immune regulation that involves the involvement of Treg cells. An understanding of the precise role of CD4+ T helper cell subsets could aid in the improved treatment of MI and prevention of its after-effects. It is worth noting, however, that I/RI is not limited to MI and reperfusion injury is also seen in cerebral, renal, hepatic, testicular, or pulmonary ischemia. Thus, when investigating the role of CD4+ T helper cells in MI/R injury, their function in I/RI of other organs should also be referenced.

### Th1 cells

Th1 cells exhibit various features, including surface markers like CD4 and CD28, as well as cytokine secretion like that of IFN-γ and tumor necrosis factor alpha (TNF-α). In general, Th1 cell development needs to be initiated in response to antigen stimulation, which is usually presented to T cells by specialized antigen-presenting cells (APC) (such as DCs). When activation of APC by PRRs, they produce significant quantities of interleukin-12 (IL-12), which stimulates IFN-γ production [42]. IFN-γ and IL-12 are pivotal cytokines in the differentiation of Naive cells into Th1 cells. IFN-γ is also capable of activating the signal transducer and activator of transcription 1(STAT1) pathway and promoting the expression of T-bet. T-bet’s function as a prominent transcription factor in the differentiation of Th1 cells is attributable to its capacity to guide differentiation and repress differentiation-linked genes in other Th cells (e.g., Th2, Th17). Further differentiation of T cells through T-bet leads to IFN-γ production, which subsequently enhances T-bet expression, creating a positive feedback loop that promotes Th1 cell differentiation [43]. The primary function of Th1 cells is to combat infections and diseases by triggering cell-mediated immune responses. These cells produce inflammatory cytokines, IFN-γ and TNF-α, and help achieve resistance to intracellular pathogens by supporting the clearance of infected cells by macrophages and CD8+ T cells. Additionally, they assist B cells in generating potent antibodies against pathogens [44]. However, excessive or inappropriate activation of Th1 cells can result in the production of inflammatory cytokines, leading to pathological outcomes.Th1 cells are widely accepted as a causative agent in organ-specific autoimmune disorders like type 1 diabetes and multiple sclerosis(MS) [45, 46]. Studies conducted in various animal models of MS have shown that Th1 cells have a significant contribution to the initiation of this autoimmune disorder [47]. Additionally, overt transfer of Th1 cells can worsen EAE [48], a mouse model of MS. The role of Th1 cells is crucial for defending against intracellular pathogens, such as Mycobacterium tuberculosis and leishmania [49]. In fibrotic diseases, Th1 cells produce IFN-γ, which inhibits fibroblasts’ collagen synthesis, leading to fibrosis attenuation [50]. Therefore, Th1 cells’ antifibrotic effects are acknowledged extensively. In a study using IL-12 to treat Schistosoma mansoni infection in mice, IL-12 induced differentiation of Th1 cells, increased expression of Th1 cytokines and suppressed Th2-dominated immune responses, and ultimately significantly ameliorated fibrosis [51]. IFN-γ increases the expression of cells’ matrix metalloproteinases (MMPs), leading to degradation of ECM components. This alteration in ECM remodeling contributes to the amelioration of fibrosis [52]. However, Th1 cells and their cytokine IFN-γ do not always exhibit antifibrotic properties. In the fibrotic myocardium of patients with nonischemic heart failure (HF), the infiltration of Th1 cells activates cardiac fibroblasts and induces fibroblast-to-myofibroblast conversion through integrin α4-dependent mechanisms. Furthermore, Th1 cells induce TGF-β expression in myofibroblasts, resulting in the formation of a fibrous ECM within the myocardium [53].

In the context of MI/R injury, Th1 cells are believed to fulfill a crucial function in the inflammatory process. Numerous studies demonstrate that following a MI, the systemic release of various cytokines and chemokines leads to activation of CD4+ T cells, which differentiate into a Th1 phenotype during the acute phase of MI/R. In a mouse model of MI/R, the levels of serum cytokines IFN-γ and TNF-α were found to be significantly elevated on the seventh day after MI/R when compared with the sham-operated group [39]. Similarly, serum levels of interleukin-6 (IL-6) and TNF-α were elevated in MI patients compared to controls on both day 1 and day 6 post-MI/R [54]. These studies suggest that these cytokines may play a role in the pathogenesis of MI/R injury. Using lymphocyte-deficient RAG1 knockout (KO) mice, IFN-γ KO mice, and wild-type (WT) controls in a mouse model of ischemia-reperfusion, researchers discovered an intriguing experimental outcome. The results demonstrated that Rag1 KO mice exhibited a decrease in myocardial inflammation and infarct size after I/R. This protective effect is reversed by reconstitution of IFN-γ-producing CD4+ T cells rather than CD8+ T cells. This indicates involvement of CD4+ T cells (Th1 cells), which produce large amounts of IFN-γ, in MI/R injury [30]. Increased Th1 cells are associated with pathological events, including the release of inflammatory cytokines, infiltration of leukocytes, and apoptosis of cardiomyocytes. Th1 cells secrete chemokines, including CCL7, and cytokines, such as IFN-γ, interleukin-2 (IL-2), and TNF-α, to promote Th1 differentiation [43]; High levels of Th1-inducing factor can inhibit the transition of M1-type macrophages to M2-type macrophages. This can lead to a reduction in healing potential after MI/R and an increase in the tissue-damaging ability of N1, Ly6Chi, and M1 cells. In addition, it promotes the recruitment of CD8+ T cells and enhances the activity of B cells, which accelerates the clearance of necrotic tissue [16]. As previously stated, CD8+ T cells and B cells play a role in amplifying inflammation and increasing tissue damage. Innate immunity involves M1 macrophages and N1 neutrophils as major contributors to I/R injury induction, while the adaptive immune system primarily serves to amplify innate immune cell and complement cascades. Th1-type immunity is crucial for injury induction.

In the normal heart, fibroblasts form a significant cell type that plays a pivotal role in preserving the heart’s structure and function. During the reparative phase post-myocardial infarction, fibroblasts become active and commence synthesizing collagen and other ECM molecules. These processes are regulated by cytokines like TGF-β, as well as potentially other cytokines and signaling pathways. Typically, interactions between Th1 cytokines and fibroblasts promote an antifibrotic response in cardiac tissue [55]. IFN-γ secreted by Th1 inhibits the pro-fibrotic activity of TGF-β, thereby impeding fibroblast proliferation and reducing the expression of type I and type III collagen mRNAs. Consequently, it acts as an antifibrotic mediator. Meanwhile, IFN-γ decreases secretion of interleukin-4 (IL-4) and interleukin-13 (IL-13) to inhibit Th2-mediated fibroblast activation and indirectly regulates fibrosis by impacting macrophage activation [56]; Furthermore, Th1 cells produce an additional antifibrotic agent called IP-10 (also known as CXCL10), which is found to elevate within 24 h of a MI [57]. The antifibrotic effect of CXCL10 is believed to result from the inhibition of fibroblast migration induced by basic fibroblast growth factor (BFGF) and the enhancement of growth factor-mediated wound contraction in collagen lattices filled with fibroblasts [58]. Overall, the presence of Th1 cells following a myocardial infarction restricts fibrosis by impeding fibroblast activation through direct and indirect means.

It may be concluded that Th1 cells are mainly linked with several negative cardiac outcomes, including heightened cardiomyocyte apoptosis, enlarged myocardial injury, disrupted ECM turnover, and reduced myofibroblast differentiation, resulting in cardiac rupture. However, the function of Th1 cells may be intricate since IFN-γ stimulates Treg differentiation that restricts Th1 and CD8 + T-cell-mediated pathology leading to cardiac healing [59]. Notably, IFN-γ levels were elevated in myocardial tissue for one week up to one month following a myocardial infarction in a mouse model [60]. In the MI/R model, IFN-γ expression in myocardial tissue persisted until day 14, with lower IFN-γ levels observed on day 14 compared to day 7 [39]. Thus, reperfusion contributed to attenuating IFN-γ-mediated inflammatory responses compared to non-reperfusion. Moreover, mice cardiac tissues after MI/R presented high levels of IL-2 cytokine and chronic inflammation, indicating the involvement of Th1 cytokine IL-2 in the chronic pathogenesis of damaged hearts after MI/R [39]. Th1 cells play a multifaceted role in MI/R by assisting in the clearance of necrotic cellular debris and promoting restoration of damaged tissue. However, they may also have a negative impact on the infarcted site, potentially leading to enlargement of the damaged area. Additionally, they inhibit fibrosis during MI repair while increasing the risk of cardiac rupture. Although the role of Th1 cells in MI/R injury has been extensively studied, additional research is needed to gain insight into their precise regulatory mechanisms and pathways of action. Such studies will help us better understand the mechanism of Th1 cell action and provide new ideas and strategies for the treatment of MI/R injury.

### Th2 cells

Cytokines IL-4 and IL-2 are crucial in Th2 cell differentiation. Major transcription factors involved in the differentiation of Th2 cells consist of signal transducer and activator of transcription 6 (STAT6) induced by IL-4 and GATA3, the primary regulator upregulated by STAT6. Different mechanisms involved in promoting Th2 differentiation by GATA3 include promoting Th2 cytokine production, selectively proliferating Th2 cells via recruitment of growth factor independent 1 (GFI-1), and inhibiting Th1 differentiation through interaction with T-bet. Furthermore, GATA3 inhibits Th1 differentiation by downregulating signal transducer and activator of transcription 4 (STAT4). GATA3 deficiency in mice shifts Naive cell differentiation towards the Th1 lineage and disrupts Th2 differentiation [43]. Th2 cells play a crucial role in regulating the humoral immune response by promoting antibody production and eosinophil activation. The significance of cytokines primarily produced by Th2 cells, specifically IL-4, IL-5, and IL-13, in promoting allergic inflammation, including human asthma and animal models of allergic respiratory inflammation, has been evidenced [61]. Th2 cells play a crucial role in eradicating parasites like Nippostrongylus brasiliensis [62]. Th2 cells also assist B cells in producing antibodies against extracellular pathogens [63]. Furthermore, Th2 cells secrete anti-inflammatory cytokines that interfere with the functioning of Th1 cells and inflammatory phagocytes [64]. Multiple studies using different experimental models of fibrosis and scar formation have demonstrated that various typical cytokines secreted by Th2 cells can promote the progression of fibrosis. Among these, IL-13, a cytokine commonly secreted by Th2 cells, has been demonstrated to stimulate fibrosis in various tissues including liver, lung, and kidney. IL-13 has been observed to promote hepatic fibrosis during the formation of liver fibrosis in Schistosoma-infected mice [65]; Overexpression of IL-13 in the lungs induces marked subepithelial respiratory fibrosis in mice without any other inflammatory stimulus [66]. Type 2 cytokines may induce the expression of key proteins involved in ECM remodeling and collagen deposition, providing a plausible mechanistic explanation [67]. Furthermore, mediators that Th2 cells secrete may impact the fibrotic process by altering macrophage phenotypic plasticity (resulting in their transformation into M2-type macrophages) [68].

It has been suggested that higher counts of Th2 cells in patient circulation could decrease the likelihood of future coronary events [69]. Studies in a mouse model of myocardial infarction revealed no notable increase in the expression level of the Th2-associated transcription factor GATA3 in the left ventricle within the initial 7 days of infarction. However, eight weeks after the occurrence of an infarction, Th2 cells become the dominant T-cell phenotype in the left ventricle and serve as crucial modulators of myocardial fibrosis and hypertrophy [70, 71]. IL-5 levels in the peripheral blood of patients with AMI are elevated within 24 h [72]. Similarly, the researchers discovered higher concentrations of IL-4, IL-5, and IL-13 proteins in myocardial tissue on days 7 and 14 of ischemia-reperfusion compared to the sham-operated group in the reperfusion model analysis [39]. Another study came to a similar conclusion that Th2 immunoreactivity, between 4 to 7 days after reperfusion of a myocardial infarction, can lead to a shift from the Th1-induced tissue damage phase of ischemia-reperfusion to a state of inflammatory regression [73]. At this stage, the amount of Th1 cells diminishes while the quantity of Th2 cells rises, enhancing the restoration effect of the Monocyte/Macrophage (M/M) population [21]. Chen et al. discovered that after cerebral ischemia and reperfusion, neural hyperexcitability worsens brain I/R injury due to IL-4 deficiency [74]. One potential explanation is that the lack of IL-4 diminishes pathways that regulate the function of microglia/macrophages, thereby impacting brain restoration [75]. Moreover, Deng et al.‘s hepatocellular I/R research indicated that IL-4 could cause hepatic macrophage M2 polarization, contingent on the STAT6-JMJD3 pathway, effectively resulting in grafts being free from I/RI post liver transplantation [76]. Removal of IL-4 and IL-13 during renal ischemia-reperfusion impedes recovery from ischemia-reperfusion injury, and correlates with a rise in M1 phenotype and a decline in M2 phenotype [77]. In a previous study conducted by Kato et al., it was observed that mice lacking IL-13 caused greater damage during hepatic ischemia-reperfusion in comparison to wild-type mice. This was accompanied by a significant increase in neutrophil accumulation on the endothelium of large hepatic veins [78]. Thus, elevated levels of IL-4 and IL-13 stemming from Th2 cells augment the quantity of reparative macrophages post-MI/R, facilitate M2-like differentiation of monocytes/macrophages, and also regulate leukocyte recruitment, ultimately enhancing wound healing within the infarcted region [79]. IL-5 production by Th2 cells enhances eosinophil proliferation required for tissue repair post-injury. Using a mouse model of MI, Xu and colleagues discovered elevated IL-5 levels in myocardial tissue after MI. Topical application of recombinant mouse IL-5 led to a marked increase in eosinophils in both the peripheral blood and the infarcted myocardium, along with reduced infarct size, increased ejection fraction, and angiogenesis in the border zone [80]. When a targeted depletion of eosinophils in mice with myocardial infarction is conducted, it can reduce the positive impact of IL-5 [80]. Mechanistic studies have demonstrated that IL-5 promotes STAT6 phosphorylation, a signal downstream of macrophages, by increasing eosinophils. This in turn leads to an increased number of CD206+ macrophages in the infarcted myocardium after MI. Conversely, this eosinophil activity is abolished by the use of IL-4-neutralizing antibodies. IL-5 facilitates recovery of cardiac dysfunction after MI by promoting eosinophil accumulation and subsequent CD206+ macrophage polarization through the IL-4/STAT6 axis [80]. Additionally, cytokines secreted by Th2 cells can inhibit Th1 responses. IL-4, in particular, induces GATA3 expression via STAT6-dependent mechanisms. This leads to a further increase in IL-4 and IL-5, while simultaneously inhibiting IFN-γ production, ultimately resulting in the inhibition of Th1 proliferation and differentiation [81]. In myocardial fibrosis, Th2 cell-secreted IL-4 and IL-13 are linked to collagen synthesis following MI. Deficiency of IL-13 hinders wound healing leading to unfavorable remodeling, whereas IL-13 enhances wound healing in infarcted regions through modulation of leukocyte recruitment and induction of M2-like monocyte/macrophage differentiation [79]. IL-4 promotes cardiac fibrosis by recruiting monocyte-derived M2 macrophages [82] and then regulating the secretion of factors such as TGF-β, collagenase, and matrix metalloproteinase, indirectly affecting cardiac fibroblasts. Notably, while Th2 responses can promote fibrosis in myocardial tissue, an excess of Th2 responses may result in pathological scarring and hyper fibrosis. Therefore, it is crucial to carefully regulate Th2 responses to ensure their beneficial role in repairing myocardial infarction. To conclude, Th2 cells play a significant immunomodulatory and reparative function in MI/R injury. They regulate immune homeostasis, activate fibroblasts, and suppress excessive inflammatory responses to aid in the repair of myocardial tissue.

### Th9 cells

Compared to other T helper cells, Th9 cells represent a newly identified subpopulation of CD4+ T helper cells. Initially classified as a Th2 subtype, Th9 cells are now defined as a distinct type of Th cell that primarily produces interleukin-9 (IL-9) [83]. TGF-β allowed for the ongoing differentiation of Th2 cells into Th9 cells, while the pairing of IL-4 and TGF-β directly caused the differentiation of naive cells into Th9 cells, and its major transcription factors are PU.1 and FoXo1 [84, 85]. PU.1 suppresses the expression of Th2 cytokines and triggers the transformation of Th2 into Th9 in vitro, contributing to the immune response by releasing IL-9. Th9 cells play crucial roles in various immune responses. They are involved in allergic inflammation, such as in cases of allergic asthma, as well as in autoimmune diseases such as rheumatic diseases, MS, ulcerative colitis, and systemic lupus erythematosus (SLE). Th9 cells also participate in immune responses to extracellular pathogens, including parasitic infections, as well as in antitumor immunity, and in inflammatory immune diseases, they perform pro-inflammatory, anti-inflammatory, and pro-tissue fibrosis functions [86–88]. Interestingly, Th9 cells, closely associated with the immunopathology of asthma, also generate IL-10 [89]. Th9 cells and IL-9-associated signaling pathways facilitate lung and respiratory inflammation by attracting and infiltrating non-specific inflammatory cells [90]. In contrast, mice lacking the IL-9 receptor exhibited phenotypic impairments in the suppressive function of CD4+FoxP3+ Treg cells when compared with WT mice, indicating a prospective protective function of the IL-9/IL-9 receptor pathway in autoimmune disorders [85]. In fibrotic diseases, Th9 cells and IL-9 play a role, with elevated IL-9 levels observed in mouse models of silicosis fibrosis as well as in patients with idiopathic pulmonary fibrosis (IPF) and cystic fibrosis. Correspondingly, the administration of antibodies that neutralize IL-9 protected mice from both IPF and cystic fibrosis [91, 92]. Similarly, IL-9 levels are markedly increased among liver cirrhosis patients and have been demonstrated to contribute significantly to the advancement of liver fibrosis. In a mouse model of liver fibrosis, IL-9 was found to activate the Raf/MEK/ERK signaling pathway. IL-9 antibodies were shown to improve hepatic fibrosis, which is consistent with studies on pulmonary fibrosis disease [93]. Th9 cells signaling through IL-9/IL-9R have a dual function in disease progression, serving both pro-inflammatory and pro-fibrotic roles while also providing protective benefits [94, 95]. In instances of tumor diseases, Th9 cells secrete the cytokine IL-9 to exert immune response on other cells, and they additionally secrete interleukin-21 (IL-21) and interleukin-3 (IL-3) to exert divergent impacts on other cells, such as DCs, cytotoxic T lymphocyte cells (CTLs), NKs, mast cells, and others. Lu et al. reported that Th9 cells are a distinct subset of CD4+ T cells, which they found to have the ability to eradicate advanced tumors through a mouse melanoma model study [96]. Shen et al. discovered that Fas signaling stimulates Th9 cell differentiation via Ca2+-dependent PKC-β activation of NF-κB in a mouse model. When Fas signaling exacerbates enteritis, Fas combined with a p38 inhibitor enhances Th9 cells’ antitumor capabilities significantly. And in patients with Non-Small Cell Lung Cancer, higher levels of Th9 cell expression were associated with a favorable prognosis [97]. IL-9 is regulated by several signaling pathways, giving it various roles. Studying Th9 and IL-9 can uncover new therapeutic targets. In general, Th9 cells seem to be pathogenic, so blocking the IL-9 pathway could be a promising approach to reducing the immunopathology of specific autoimmune and inflammatory diseases. However, as previously mentioned, Th9 cells also produce the powerful anti-inflammatory cytokine IL-10 and may serve immunomodulatory roles, in addition to their function in eliminating cancer and bacterial pathogens.

IL-9 and related cytokines were detected in the ischemic brain and blood following transient focal cerebral ischemia in rats. In the experimental group, IL-9 levels were significantly elevated after middle cerebral artery occlusion compared to the sham-operated group [98]. Similarly, the researchers discovered that following AMI, Th9 cells and plasma IL-9 levels were increased, accompanied by the rise of the major transcription factor PU.1 expression [99, 100]. In a mouse model of reperfusion injury, the protein expression level of IL-9 significantly increased in cardiac tissue on both days 7 and 14 following MI/R. However, there was a significant decrease in the expression level of this cytokine on day 14 compared to the day 7 group [39]. This study indicates that Th9 cells may play a role in the inflammatory response following infarct reperfusion and persist in the damaged heart until day 14, leading into the chronic phase. Complement activation is recognized as a significant pathogenic mechanism of reperfusion injury. According to a study by Hu et al., the reduction of cytokine levels such as IL-9 by knocking out complement-related genes in mice attenuated pathological injury and inhibited apoptosis after renal reperfusion, indicating a correlation between reperfusion injury caused by the complement cascade and IL-9 [101]. The pathophysiology of I/R injury is regulated by the inflammatory response. Kortekaas evaluated the role of IL-9 in renal I/R injury and found a different conclusion. Donor grafts release large amounts of IL-9 during reperfusion in clinical kidney transplantation. Worsened renal injury was observed with experimental inhibition of these IL-9, suggesting that IL-9 plays a regulatory role in clinical I/R injury [102]. IL-9 has been demonstrated to increase the killing activity of CD8+ T cells, potentially by reducing CD8+ T-cell depletion. This is accomplished by up-regulating the production of perforin and granzyme B while downregulating the expression of PD-1 and CTLA-4, effectively inhibiting immune checkpoints [99]. Suggests that heightened secretion of IL-9 by in vivo CD4+ T cells (Th9 cells) in patients with AMI increases the cytotoxicity of CD8+ T cells, thereby exacerbating cardiomyocyte injury. In MI/R injury, Th9 cells and their associated cytokine IL-9 play significant roles in amplifying the inflammatory response and injury. The Th9 cells primarily mediate the inflammatory response following MI and the expression level of IL-9 is positively correlated with the severity of injury. The interaction between complement activation and IL-9 regulatory function is also involved in reperfusion injury. These results are significant for comprehending the development of reperfusion injury or devising therapeutic approaches.

### Th17 cells

Th17 cells are a subset of helper T cells, and the differentiation of Th17 cells is mainly driven by cytokines like IL-6, IL-21, interleukin-23 (IL-23), and TGF-β. The transcription factors responsible for regulating Th17 cell differentiation are RORc in humans and RoRγt in mice. The differentiation process can be categorized into three stages: a differentiation stage mediated by TGF-β and IL-6, a self-amplification stage mediated by IL-21, and a stabilization stage mediated by IL-23 [43]. The self-amplification phase plays a critical role in the differentiation process as it is crucial for creating a robust immune response. Unlike the mechanism underlying Th1 and Th2 differentiation, where the respective major cytokines, IFN-γ and IL-4, can act as the corresponding amplifying cytokines, the major cytokine, interleukin-17 (IL-17), of Th17 cells does not promote their differentiation. In contrast, the substantial production of IL-21 by Th17 cells collaborates with TGF-β to amplify Th17 differentiation [103]. IL-23 production by antigen-presenting cells (APCs) promotes the third phase, providing significant fundamental knowledge for examining the function of Th17 cells in cardiovascular disease. Th17 cells are pivotal immune cells that have garnered significant interest due to their extensive involvement in various diseases. They play a crucial pathogenic role in ailments such as inflammatory bowel disease (IBD) and MS [104, 105]. Meanwhile, Th17 cells have also been linked to the development of rheumatoid arthritis (RA), which may result from the dysregulation of the Treg and Th17 balance principle [106]. Furthermore, Th17 cells can boost the development of ocular diseases like dry eye disease by directly enhancing the proliferation, differentiation, and plasma cell production of B cells [107]. Th17 cells secrete a range of cytokines, notably IL-17A and interleukin-17F (IL-17F), IL-22, and IL-21. Dysregulated levels of Th17 cytokines correlate with disease activity and severity in patients with SLE [108]. While the Th17 cytokine IL-17A has been implicated in the pathogenesis of psoriasis, psoriatic skin displays elevated expressions of IL-17A and IL-17F, which stimulate immune and non-immune cells and incite tissue inflammation. IL-17A and IL-17F play key roles in neutrophil accumulation and the subsequent formation of epidermal microabscesses in psoriatic lesions [109]. In cases of neutrophilic asthma (NA), the Th17 cytokine, IL-17, causes significant neutrophil infiltration in the lungs, resulting in pathogenic effects [110]. Furthermore, dysregulation of IL-22 production by Th17 cells has been linked to a variety of inflammatory skin diseases, including psoriasis, atopic dermatitis (AD), and allergic contact dermatitis (ACD) [111]. Th17 cells are known for their high production of IL-17 and significant contribution to the inflammatory response [112]. The capacity of IL-17A to cause endothelial, epithelial, and stromal cells to generate diverse pro-inflammatory mediators including interleukin-1 (IL-1), IL-6, TNF-a, CXCL8, granulocyte colony-stimulating factor, and granulocyte-macrophage colony-stimulating hormone is likely to result in the recruitment and activation of neutrophils [113]. Additionally, Th17 cells have the capability to chemotactically attract neutrophils and stimulate inflammation via the release and production of CXCL8 [114]. Th17 cells are crucial pathogenic factors in fibrotic diseases, in addition to inflammation. A study conducted on mice with hypersensitivity pneumonitis revealed that diminishing IL-17R lessened both inflammatory and fibrotic responses [115]. In recent years, there have also been a number of studies showing a strong association between Th17 cells and liver fibrosis. Among them, increasing the proportion of CD4+ IL-10+ T cells can reduce liver fibrosis by reducing the number of Th17 cells that produce IL-17 [116]. In the liver, IL-17 acts on multiple cell types, including Kupffer cells and hepatic stellate cells (HSCs). Previous studies have shown that IL-17 activates the relevant mechanism of STAT3 pathway in hsc, thereby promoting the synthesis of type 1 collagen [117]. In addition, in the area of cardiac fibrosis, IL-17A signaling to cardiac fibroblasts leads to induction of granulocyte-macrophage colony-stimulating factor (GM-CSF) and CCL2, which drives Ly6Chi monocytes to chemotaxis and accumulate in the heart, worsening dilated cardiomyopathy outcomes [118]. These findings reveal the important role of Th17 cells in inflammatory injury, hepatic fibrogenesis and myocardial fibrosis and provide an important reference for further understanding the role of Th17 cells in disease pathogenesis. Th17 cell recruitment has been observed in a variety of malignant tumors compared to healthy tissues, but the exact mechanism of recruitment is unknown and its impact on overall disease prognosis is variable. IL-17 cytokines are associated with increased vascularization and thus increased tumor growth and metastasis in some models, and transdifferentiation of Th17 cells into T cells with a more immunosuppressive phenotype plays a role in tumor immune evasion [119]. However, recruitment of CD8+ cytotoxic T cells and DCs to the tumor site by Th17 cells promotes tumor clearance, similar to their ability to convert to an IFN-γ-secreting Th1 phenotype in response to certain environmental factors [119]. Overall, the role of Th17 cells in cancer progression appears to be highly dependent on the specific tumor microenvironment. Exploiting this plasticity to control them to enhance antitumor responses may be a useful strategy for the development of cancer immunotherapy.

In hepatic I/R injury, IL-17A is a key regulator of the initiation of neutrophil-induced inflammatory response and liver injury in the subacute phase after reperfusion [120]. Study shows significant increase in Th17 cells in AMI patients [121]. And 8 weeks after infarction, Th17 cells became one of the predominant T-cell phenotypes in the left ventricle, suggesting that they may play an important role in the process of chronic cardiac remodeling [71]. Similar to IL-9 described previously, protein expression levels of IL-17 were significantly higher in cardiac tissue on days 7 and 14 after MI/R in mice, but expression levels of this cytokine were significantly lower in cardiac tissue on day 14 compared to the day 7 group [39]. These results indicate that the inflammation in the injured heart mediated by IL-17 continues until day 14, at which point it transitions to the chronic phase. As previously noted, Th17 cells may contribute significantly to heart damage by inducing or boosting the expression of inflammatory cytokines, including IL-6, IL-1β, and TNF-α, resulting in an increased inflammatory response that causes chemotaxis and activation of neutrophils in the damaged heart. This exacerbates the inflammatory response, causing more damage to cardiomyocytes. Reducing cardiomyocyte apoptosis and neutrophil infiltration while mitigating left ventricular dilatation on day 28 post-MI improved survival in the absence of IL-17 [122]. IL-21, a cytokine commonly produced by Th17 cells, is a valuable biomarker in predicting myocardial function following an AMI [123]. As previously discussed, Ly6Clow macrophages facilitate scar formation and impede infarct expansion in the early stages of MI. Furthermore, Ly6Clow macrophages demonstrated heightened expression of IL-21R compared to Ly6Chigh macrophages. Indeed, CD4+ T cells extracted from infarcted hearts produce IL-21 upon stimulation. Furthermore, IL-21-deficient mice exhibited a shorter duration of inflammation post-MI event and experienced significantly better survival and cardiac function in comparison to WT mice. Consistent with this, IL-21-deficient mice exhibited a notable decrease in neutrophil counts and MMP-9 levels, alongside a significant rise in Ly6Clow macrophage numbers. Further research found that IL-21 increases apoptosis in Ly6Clow macrophages. Conversely, neutralizing the IL-21 receptor Fc protein usage enhanced the amount of Ly6Clow macrophages in infarcted hearts, leading to improved survival rates and cardiac function in individuals who recently experienced a MI. Thus, IL-21 slows down the healing process of wounds by causing cell death in Ly6Clow macrophages, leading to decreased survival rates following MI [124]. IL-21-secreting cells play a role in the pathology of I/R injury. Additionally, the IL-21 signaling pathway may contribute to the I/R injury process in allografts, resulting in elevated IL-21 expression within the grafts [125]. T lymphocytes play a pivotal role in the acute phase of I/R injury. In a study using a mouse model of transient focal cerebral ischemia, Clarkson et al. discovered greatly elevated levels of IL-21 in the brains of mice that suffered from cerebral ischemia. Furthermore, targeted deletion of IL-21 led to a reduction in infarct size and improved neurologic function in mice. The IL-21 was derived from CD4+ T cells that infiltrated the brain. Therefore, CD4+ T-cell-derived IL-21 directly correlates with cerebral I/R injury [126]. Similarly, it is likely that Th17 cells were involved in the acute phase of MI/R injury, as CD4+ T cells were identified as the primary source of IL-21, which was found to be elevated. Myocardial injury worsened significantly following exogenous IL-21 treatment, which led to an increase in neutrophil infiltration. Conversely, neutralization of IL-21 led to a reduction in MI, resulting in a decrease in infarct size and an improvement in cardiac function. In-depth research findings have shown that the typical cytokine IL-21 promotes neutrophil infiltration by inducing the expression of two neutrophil chemokines (CXCL1 and CXCL2) in cardiomyocytes through the Akt/NF-κB and p38MAPK/NF-κB signaling pathways. Furthermore, experiments involving the depletion of neutrophils demonstrated a reduction in myocardial injury, which supports the fact that IL-21 aggravate myocardial injury primarily by increasing neutrophil infiltration [127]. In addition to their role in the inflammatory response, Th17 cells may promote ECM remodeling after myocardial injury by producing repair-related components, including MMPs or proteoglycans. However, an excess of Th17 cells may result in excessive ECM remodeling, which can lead to adverse left ventricular remodeling [128, 129]. Thus, the complexity of the prognostic impact of Th17 cells is complex. Notably, a separate investigation demonstrated that patients with AMI and reduced serum IL-17 levels were substantially associated with an increased risk of major cardiovascular events [130]. The evidence indicates that the mechanism of action of Th17 cells involving the cytokine IL-17 may not be fully understood, and additional investigations are required to explore its function in reperfusion injury following MI/R. While studies on the functionality of IL-21 are relatively complete, targeting IL-21 may have therapeutic potential in MI/R injury. It should be noted that IL-17 in the post-MI setting may stem from sources other than Th17 cells, and therefore, some of the findings may not accurately reflect the influence of Th17 cells.

### Th22 cells

Th22 cells are a subset of T helper cells that secrete IL-22 and TNF-a but not IL-17 or IFN-γ [131]. This distinguishes Th22 cells from other Th cells. Th22 differentiation is induced by IL-6 either alone or in combination with TNF-α. Its transcription factor AHR and is regulated by the transcription factor RORc [111]. Th22 cells express chemokine receptors CCR4, CCR6, and CCR10 [132]. IL-22 is a crucial cytokine for the functioning of Th22 cells, and it belongs to the IL-10 cytokine family. Both Th22 cells and IL-22 are evident in numerous diseases. MS is an autoimmune, inflammatory disease caused by the recruitment of self-reactive lymphocytes in the central nervous system. In the beginning, research indicated significant alterations in the frequency and mechanisms of Th22 in MS patients [133]. Subsequently, further studies determined that Th22 cells act primarily on astrocytes through IL-22 release [134]. This could potentially have a harmful impact on MS by increasing permeability of the blood-brain barrier through the secretion of MMPs [135]. Psoriasis and IBD are closely linked inflammatory conditions. Previously, they were considered Th1-linked conditions. However, new T-cell populations, such as IL-22 and Th22 cells, have recently been highlighted due to their potential role in psoriasis and IBD [136]. IL-22 acts on skin cells to worsen psoriasis inflammation [137]. In IBD, a damaged epithelial barrier causes and sustains the disease. IL-22 promotes tissue regeneration, strengthens immune response, and produces mucus, making it a possible treatment for healing the gut lining in IBD patients [138]. In a study of lung injury caused by bleomycin, researchers discovered that Th17 cells produce most of the IL-22 and IL-17. Airway inflammation improved when IL-22 was blocked during bleomycin administration, indicating that IL-22 has pro-inflammatory effects. However, in mice without IL-17, their T cells still produced high levels of IL-22, and airway inflammation significantly decreased. This suggests a protective role for IL-22 in the absence of IL-17 [139]. Thus, the role played by Th22 cells in airway disease is uncertain. Th22 cells have a significant role in causing allergic airway diseases. They increase IL-22 secretion and modulate immune system pathways, resulting in pro-inflammatory effects through activation of IL-5, IL-13, interleukin-33 (IL-33), and TGF-β1. Th22 also has anti-inflammatory properties, reducing pro-inflammatory cytokines and eosinophils through its action on IL-10 [140]. Th22 cells are linked to the cause of RA. A study conducted by Zhong et al. reveals that Th22 cells in circulation and plasma IL-22 levels worsen RA. Using a combination therapy of MTX + LEF to decrease IL-22 levels and target Th22 cells can remedy immunodeficiency, eventually improving RA symptoms [141]. While IL-22 can be produced by numerous cell types, Th22 cells are a principal source of IL-22 during the latter stages of inflammation. This implies that the Th22 cells may have a vital role in regulating chronic inflammation [142]. Th22 cells have been found to have an antiviral role in human viral infectious diseases. HIV infection caused a significant reduction in IL-22-producing Th22 cells, leading to impaired epithelial integrity and increased microbial translocation. The administration of recombinant IL-22 showed a protective effect against HIV-induced intestinal epithelial damage. Therefore, the study’s findings suggest that the reduction of IL-22 secretion and Th22 depletion in the intestinal mucosa play a significant role in the pathogenesis of HIV mucosal immunity [143]. In hepatitis B virus (HBV) infected individuals, IL-22 can activate the production of liver stem/progenitor cells (LPC) by activating the STAT3 pathway, achieving a protective role in HBV infection [144]. IL-22 is involved in the development of tumor diseases, the excess presence of IL-22 in the tumor microenvironment promotes tumor growth, inhibits apoptosis, and facilitates metastasis via STAT3 pathway activation [145].

IL-22 also plays a crucial function post-MI. In humans, Th22 cells are primarily responsible for producing T cells that secrete IL-22 [146]. In a study conducted by Zhang and colleagues, it was discovered that the ratio of Th22 cells to CD4+ IFN-γ-cells was significantly higher in patients with AMI compared to healthy individuals. Plasma IL-22 levels were significantly higher in AMI patients. Additionally, Th22 cells showed a positive correlation with plasma IL-22 levels among AMI patients [121]. Similarly, Lin and colleagues demonstrated a notable elevation in Th22 cells among patients after AMI compared to healthy controls. They also observed a significant increase in AHR expression and upregulated IL-22 expression in peripheral blood after 12 h [100]. It was seen that after the onset of AMI, the percentage of Th22 cell subsets increased and the associated cytokine IL-22 was also positively elevated. A study conducted by Tang et al. found that administering subcutaneous injections of IL-22 to mice for 7 consecutive days after MI significantly improved their rate of survival. Additionally, the group treated with IL-22 showed a significant decrease in infarct area and collagen volume fraction on day 28, compared to the saline-treated control group [147]. IL-22 treatment inhibited the infiltration of total leukocytes, neutrophils, monocytes/macrophages, NK cells, γδ T cells, and B cells, which correlates with poor prognosis. Conversely, IL-22 treatment promoted the infiltration of αβ T cells [147]. Thus, the therapeutic benefit of IL-22 may result from its ability to inhibit leukocyte accumulation, leading to improved cardiac function post-myocardial infarction in mice. Previously, IL-22 has exhibited tissue protection and repair enhancement by activating STAT3 during inflammation in various organs [148, 149]. Thus, further research uncovered that the hepatocyte-induced activation of STAT3, as opposed to cardiac cells, is responsible for the cardioprotective effect of IL-22 post-MI by raising FGF21 levels [147]. In a separate study utilizing a reperfusion model of MI, researchers proposed that IL-22 exhibited a protective effect. However, conflicting opinions on this matter exist. The researchers drew the conclusion that IL-22 acted directly on cardiomyocytes due to the significant increase in IL-22R1 expression in cardiac tissues compared to normal mouse leukocytes. Moreover, this expression was mainly observed in cardiomyocytes. The findings indicate that the upregulation of IL-22 receptor IL-22R1 protein expression occurred after I/R, and that IL-22 activated the myocardial STAT3 signaling pathway in vivo. Additionally, the expression of P-P53/P53 (apoptosis factor) was significantly reduced in myocardial tissues after I/R injury. These results suggest that IL-22 exhibits a protective effect against myocardial injury and apoptosis via the IL-22-IL-22R1-STAT3-P53 axis [150].

The role of Th22 cells in cardiac fibrosis after reperfusion following MI remains unexplored. However, their role in post-inflammatory fibrosis in various organs can be compared. Th22 cells are involved in protecting the liver from the consequences of fibrosis in hepatic fibrosis [151]. Th22 cell levels showed elevation in CCl4-induced hepatic fibrosis mice [152], methionine choline deficiency (MCD) diet-induced nonalcoholic steatohepatitis (NASH) mice [153], and in patients with human liver cirrhosis (LC) [154]. Hepatic infiltrating Th22 cells and their cytokine IL-22 can attenuate hepatic stellate cell activation, achieving protective effects and ameliorating fibrosis [152]. Similarly, in a mouse model of chronic myocarditis and dilated cardiomyopathy induced by coxsackievirus B3 (CVB3), the study found increased levels of Th22 and IL-22, along with upregulation of MMP-9 expression and downregulation of metalloproteinase inhibitor-1 (TIMP-1) expression [155]. Additionally, IL-22-neutralizing antibodies worsened myocardial fibrosis and increased mortality. This exacerbation of fibrosis could be attributed to the Th22 cells’ potential to discharge various fibroblast growth factor family members, such as FGF1, FGF5, FGF12, and FGF13, which primarily control wound healing, tissue recovery, tissue renewal, and fibrotic processes, in addition to IL-22 [156]. In conclusion, while Th22 cells play a role in the protection of liver and cardiac fibrosis, their involvement in cardiac fibrosis triggered after reperfusion of myocardial infarction remains unexplored and necessitates further research.

### Other CD4+ Th cells

Th3 cells are characterized by the secretion of TGF-β and IL-10 and the expression of FOXP3 [157]. LAP, a membrane-bound prepeptide, binds TGF-β and Helios, and has been identified as a th3 cell marker [158]. Upon induction of TGF-β, naive CD4+ T cells can differentiate into Th3 cells, and a large number of studies have described their important role in oral tolerance to non-self-antigens and in reducing autoimmune responses [159]. Th3 cells are considered a subpopulation of inducible regulatory T (iTreg) cells [160]. They collaborate with other regulatory T cells, including nTreg cells, to prevent the immune system from launching an overzealous assault on its own tissues while also safeguarding against foreign pathogen invasion. It has been demonstrated that enhancing iTreg cells decreases the damage caused by liver I/R [161]. The anti-inflammatory role of TGF-β and IL-10 in reperfusion injury, as represented by their typical cytokines, has been extensively documented [162]. IL-10 is essential in maintaining tissue equilibrium during both infection and inflammation. It achieves this by curbing excessive inflammatory reactions, enhancing innate immune responses, and promoting tissue repair mechanisms [163]. The targeted delivery of IL-10 to ischemic AKI was investigated to modulate macrophage phenotype and abrogate inflammation [164]. TGF-β is a cytokine possessing a variety of immunomodulatory functions. Its roles in the immune system comprise the suppression of immune responses, the reduction of activated T-cell activation and proliferation, and the promotion of Treg cell formation and function [165]. M2 macrophages and other cells also inhibit inflammation after a MI through the release of TGF-β and IL-10 [166]. Because there has been a limited number of studies examining Th3 cells in isolation concerning MI/R, a myriad of unanswered questions exists pertaining to their properties and functions. More research is necessary to obtain a comprehensive understanding of Th3 cells and their involvement in infarct reperfusion.

Another subpopulation of CD4+ T helper cells known as Th25 can produce IL-25, which has been shown by numerous studies to play a crucial role in triggering an allergic response by connecting the innate and acquired immune responses in asthma [167]. Interleukin-25 (IL-25) can enhance an anamnestic inflammatory response by affecting other types of cells [167]. IL-25, also referred to as IL-17E, was initially discovered as a secretion of Th2 cells and has been subsequently found to be secreted by Th25 cells. This secretion is responsible for enhancing type 2 response through the activation of signal transducer and activator of transcription 5 (STAT5), and leading to increased production of IL-4, IL-5, and IL-9 [168]. For example, the overexpression of IL-25 in a mouse model result in airway eosinophilia and increased serum levels of cytokines IL-4, IL-5, and IL-13, which are typical of Th2 cells. Additionally, there is a rise in serum titers of IgE [169]. By contrast, IL-25-deficient mice demonstrate decreased Th2-type immune responses [170]. Moreover, blocking IL-25 via sIL-25R prevents experimental asthma in mice by inhibiting the development of AHR, allergen-specific IgE production, allergic airway inflammation, and increased mucus production [171]. Tamachi and colleagues demonstrated that IL-25 exacerbates allergen-induced airway inflammation by amplifying pre-existing Th2-type immune responses [172]. In cases of tumor diseases, IL-25 has been found to suppress tumor growth in mice that have been transplanted with different tumor cell lines. However, the antitumor effect of IL-25 was not observed in server combined immune-deficiency (SCID) mice but was observed in nude mice, suggesting the involvement of B-cell activation that is mediated via IL-17E [173]. Research on the role of IL-25 in reperfusion injury is limited because studies on IL-25 have concentrated on its connection with allergy, immunomodulation, and tumor immunity. While the role of IL-25 in reperfusion injury has not been thoroughly examined, other interleukin family members, including IL-17A and IL-17F, have been identified as playing a role in reperfusion injury. These cytokines may influence the inflammatory response while reperfusion injury progresses, affecting the severity of injury and the recovery process by recruiting and activating immune cells. It should be noted that further discoveries about the role of IL-25 and Th25 in reperfusion injury are anticipated as research advances.

**Fig. 3:**
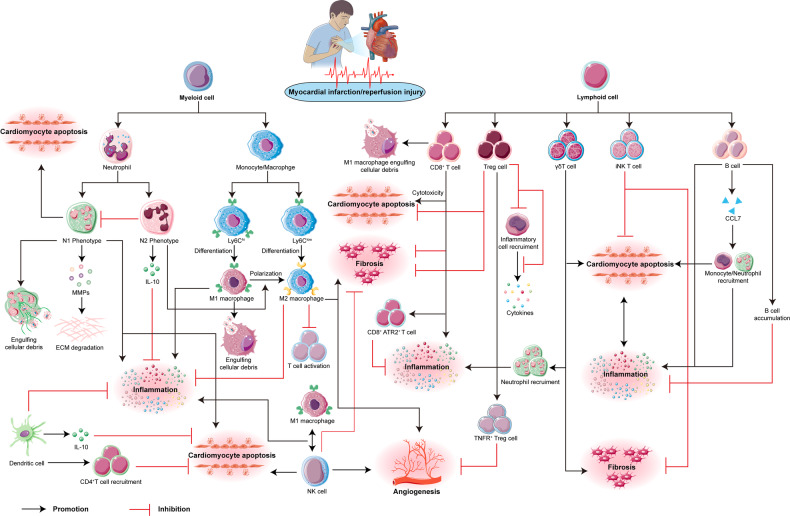
General overview of the role of CD4+ T helper cell subsets after reperfusion in myocardial infarction. The figure demonstrates the responses of Th1, Th2, Th9, Th17, and Th22 cells to reperfusion after myocardial infarction. This includes their direct effects on the infarcted myocardium or indirect effects on post-infarction myocardial tissues through interactions with other leukocytes. The figure also shows the mutual influences and interactions of the subsets in MI and reperfusion injury.

In summary, we’ve explored the roles of different Th cell subsets post-MI. Figure 3 visually captures the nuanced contributions of Th cells, complementing our detailed textual discussion.

## Single-cell sequencing in MI

The advances in single-cell sequencing technology have provided new perspectives and tools for MI research. Single-cell sequencing has enabled the identification of cellular heterogeneity, cell-cell interactions, and dynamic changes in cellular status in cardiac tissues following MI with single-cell resolution. By analyzing the transcriptomic profiles of peripheral blood mononuclear cells through single-cell RNA sequencing, the research team was able to identify 27 distinct cell clusters. These included monocytes, T cells, NK cells, B cells, megakaryocytes, and CD34 cells [174]. Further studies of CD4+ Th cells after MI revealed that the myocardial and lymph node environments contributed to the differentiation of antigen-specific T cells into Tregs with suppressive functions. These Tregs exhibited anti-inflammatory properties and promoted myocardial repair and functional improvement [175]. Additionally, single-cell T-cell receptor sequencing has been employed to ascertain the provenance of cardiac Tregs. It was determined that Tregs in the heart are primarily recruited from circulating Tregs and exhibit significant clonal expansion in the damaged myocardium [34]. In damaged heart muscle, the researchers employed single-cell sequencing technology to ascertain the impact of CD4+ T-cell elimination on cardiac fibrosis and cardiomyocyte proliferation. In contrast, CD8+ T cells did not appear to play a significant role in this process. These findings underscore the differential regulatory role of different T cells in cardiac regeneration and repair during development [176]. Through single-cell sequencing technology, not only can the immune characteristics of MI be revealed, but also prognosis-related markers can be identified. This approach provides a novel understanding of the molecular characteristics and mechanisms of MI at the single-cell level, thereby expanding the knowledge of the pathological process of MI [177]. Single-cell sequencing technology has not only provided insight into the complexity of cellular heterogeneity and cell-cell interactions after myocardial infarction, but has also revealed the role of key immune cells in cardiac repair. As technology continues to advance and research continues to deepen, it is hoped that single-cell sequencing will improve the understanding of Th cell role after MI/R and significantly improve the prognosis of patients with MI.

## Conclusion

MI and reperfusion injury are multifaceted and ongoing processes, in which CD4+ Th cells play a critical role. This review aims to explore the specific roles and mechanisms of CD4+ Th cells in immune-inflammatory responses and cardiac remodeling. Studies indicate that CD4+ Th cells significantly influence cardiac function and prognosis. CD4+ Th cells primarily impact three major events following myocardial infarction: inflammation, anti-inflammation, and remodeling/repair, exerting either promoting or inhibiting effects (summarized in Table 1). Specifically, different Th cell subsets play distinct roles in these events, Th1 cells enhance inflammation by promoting the clearance of cellular debris and antifibrotic processes, while also exacerbating myocardial injury. Th2 cells facilitate myocardial tissue repair by modulating immune balance, activating fibroblasts, and suppressing excessive inflammatory responses. Th9 cells increase the cytotoxic activity of CD8+ T cells, intensify inflammation and damage, and are involved in complement cascade reactions. Th17 cells augment neutrophil infiltration, promote inflammatory responses, exacerbate myocardial damage, and contribute to matrix remodeling following myocardial injury through the production of MMPs or proteoglycans. Th22 cells improve cardiac function post-myocardial infarction by producing IL-22, which inhibits leukocyte accumulation and modulates inflammation. However, several critical questions remain to be addressed, including why the same subtype of CD4+ Th cells exhibit different functions, how to identify the primary contributors when multiple cells express the same cytokines, and how to effectively regulate CD4+ T-cell functions to mitigate cardiac injury. Regulating the functions of CD4+ helper T cells requires the coordinated action of various cell types, making this task challenging and directionally uncertain. Achieving comprehensive regulation of the entire immune response necessitates a systemic, holistic, and integrative approach. Exploring and modulating key immune cells can lead to effective control of the overall immune response, ultimately protecting the myocardium. Further research and analysis are essential to address these issues, which will aid in the development of more effective therapies to reduce injury following MI.

**Table 1:** Summary of Th Cell Types: Classification, Surface Markers, Origins, and Functions.

Th cells classification	Surface marker	Function after MI	Origins
Th1	CD3,CD4,CD119,CD183(CXCR3),CD195(CCR5)…	Exacerbates the inflammatory response, leading to cardiomyocyte apoptosis and tissue damage, while also facilitating the process of cell debris removal [43]. Inhibited the production of reparative macrophages and enhanced the activity of other immune cells, contributed to the exacerbation of myocardial reperfusion injury [16]. Inhibition of cytokines such as TGF-β, IL-4, and IL-13 restricts fibroblast activation and collagen synthesis, thereby reducing fibrosis after myocardial infarction [56]. The concomitant production of IP-10 also has an antifibrotic effect [58].	Naive T cells
Th2	CD3,CD4,CD119,CD193(CCR3),CD194(CCR4),CD365(Tim-1)…	Promotes myocardial tissue repair by modulating macrophage phenotype, inhibiting inflammatory cells and thereby suppressing excessive inflammatory response, and increasing proliferation of repair-related cells [79, 80]. Promoting fibrosis in injured myocardium may lead to pathological scarring and hyper fibrosis [82].	Naive T cells
Th9	CD3,CD4,TCR α/β…	Complement cascade-induced reperfusion injury is associated with IL-9 [101]. Regulatory role in clinical I/R injury [102]. Enhancement of killing activity of CD8 + T cells to enhance inflammatory response and injury [99].	Naive T cells
Th17	CD3,CD4,TCR α/β, IL-23R, CD194(CCR4),CD196(CCR6)…	Increased neutrophil infiltration promotes inflammatory response, exacerbates myocardial injury [122]. Causing cell death in Ly6Clow macrophages to slow the wound healing process leads to decreased survival after myocardial infarction [124]. Promoting matrix remodeling after myocardial injury by producing repair-related components such as MMPs or proteoglycans [128, 129].	Naive T cells
Th22	CD3,CD4,TCR α/β,CD194(CCR6),CD196(CCR6),CCR10…	Inhibition of leukocyte accumulation and modulation of the inflammatory response leads to improved cardiac function after myocardial infarction [147]. Direct inhibition of myocardial apoptosis [150].	Naive T cells
Other:Th3	CD3,CD4,CD69,LAP,CD122…	May inhibit excessive inflammatory response and reduce inflammatory damage to cardiac tissue. Promote tissue repair to reduce pathological changes after myocardial infarction [162].	Naive T cells
